# 

**Published:** 1892-01-16

**Authors:** 


Notices of **Births, Deaths, and Marriages**, are inserted in this column at a charge of 2s. 6d. for an announcement not exceeding four lines.

### NOTICE TO CORRESPONDENTS.

All MS., Letters, Books for Review, and other matters intended for the Editor should be addressed The Editor, The Lodge, Porchester Square, London, W.

The Editor cannot undertake to return rejected M.S., even when accompanied by stamped directed envelope.

**Notice to Advertisers and Subscribers.**

All Advertisements, Orders for Copies of The Hospital, and Business Communications should be addressed to The Publisher, *not to the Editor*, The Hospital, 140, Strand, W.C.

The Cost of Subscription, post free, is as follows:—Per week, 1½d.; per quarter, 1s. 8d.; per half-year, 3s. 3d.; per annum, 6s. 6d. Foreign:—Per annum, 8s. 8d.; payable, in all cases, in advance.

**"Burdett's Hospital Annual," 1891—1892.**

Third year of publication. 600 pp. Boards, gilt lettering. A most complete guide to British and Colonial Hospitals, Dispensaries, Nursing and Convalescent Institutions, and Asylums. Price 3s. 6d. Published by The Hospital (Limited), 140, Strand, W.C.

**NOTICE.—The Index for Vol. X. (ending September, 1891), is on sale at the Office of this Journal, price 2½d., post free.**

### GENERAL CHARITIES.

[This Column is devoted to an account of work of charitable institutions other than Medical Charities. The Editor will be glad to receive reports or news of work at 140, Strand. *Philanthropy* should be plainly written on the envelope.]

A true lover of animals sent a special donation to the funds of the Home of Rest for Horses in order that the animals might open the New Year with a good meal of carrots, bread, sugar, and apples.

It was a kindly thought which prompted Sir Augustus Harris to invite the whole of the children in the Post Office Orphan Homes to see his pantomime at the Theatre Royal, Drury Lane. The visit will form a red-letter day in their calendar of joys, and their enjoyment of such a treat would be a refreshing sight.

An appeal, endorsed by Princess Louise, Marchioness of Lorne, has been issued on behalf of the **NATIONAL INDUSTRIAL HOME FOR CRIPPLED BOYS**, Wright's Lane, Kensington. Though there is accommodation for 100 boys, and a great pressure of candidates for admission, the Committee cannot receive more than 85 until the mortgage, with which the home is incumbered has been paid off. This mortgage has recently been considerably reduced, but £2,000 still remains to be paid off before the full number of boys can be admitted to the benefit of the institution.

The Bishops of Norwich and Southwark, and the Bishop Designate of Carlisle have become Vice-Presidents of the **SOCIETY FOR WAIFS AND STRAYS.** It was unfortunately necessary for the Executive Committee to call the attention of the public at their meeting last week to the necessity for increased support to enable the Society to carry on its work of rescuing waifs and strays. Owing to a considerable deficit in the funds of the Institution the number of cases to be entertained has at present to be limited. It is, therefore, to be hoped that the public will respond to the call, in order that the number of those now in the homes or boarded out under the care of the Society may not be reduced.

The Rev. Father Nugée, of St. Austin's, New Kent Road, appeals for help to support the movement for giving free breakfasts to unemployed men and women and workgirls. He defends himself from the charge of encouraging imposters and loafers in a manner which savours somewhat of the Emerald Isle. Like Aristotle, he claims that he gives not to the man but to humanity, and that he would rather help a large number and find he had been taken in ten times than close his purse, and no doubt the public's, because he is sometimes deceived. This is but a humane sentiment, to take exception to which would seem hard-hearted. There is no doubt that it is impossible to do any good without helping some undeserving cases.

Those who are interested in the work of the **MISSION TO DEEP SEA FISHERMEN** cannot do better than purchase a copy of this month's "Toilers of the Deep," in which they will find an interesting account of the work done lately by the mission: work which has been rendered doubly arduous during the storms of the present winter and past autumn. It is satisfactory to know that a generous response has been made to the appeal issued in December last for increased help for the general funds; and it is to be hoped the society will receive continued and not spasmodic help, especially with a view to the addition of the *Alice Fisher* to the Mission Fleet, and to the extension of the service in Newfoundland as suggested in an excellent letter from Mr. F. J. S. Hopwood in this month's number of "Toilers of the Deep."

Some useful information upon the progress made in the management of our poorhouses during the past twenty years is to be gleaned from a small pamphlet, entitled "Twenty Years in a Poorhouse." The system or want of system, which prevailed twenty years ago has undoubtedly been vastly improved, but it is clear that abuses still remain to be rectified. The sanitation and treatment of the sick have received at last the attention they deserve, and a poorhouse is no longer a mass of poor people cooped in together promiscuously, but has become a well-organised institution, where humanity has taken the place of brutality. Mr. Dalgleish, the author of the pamphlet, looks forward, we trust not too sanguinely, to an improvement during the next twenty years even greater than during the period of which he treats, and to a time when the rates will be reduced to a mere fraction in the pound. Happy the people who are born in those latter days!

We have received a report of the work done by **THE ORPHANS' HOME**, Austral Street, West Square, S.E. The main object of the institution, which started twenty-four years ago, is to care for the poorest and most friendless orphan children. There are at present over two hundred such children in the Home, who are taught a plain English education and receive domestic training. Those who are waiting for admission are taken as far as possible in rotation, but no orphan is received who is not destitute. We venture to think that the balance-sheet might be improved by giving more details of the expenditure, instead of classifying several important items under one heading. It would be interesting to know amongst how many matrons and teachers the sum of £630 is divided as salaries, and what the seaside expenses consisted of. It is impossible to tell from the balance-sheet, as now drawn up, how much is expended in postages and how much in printing.

### SCRAPS AND GLEANINGS.

THE Monmouth General Hospital has done a good year's work, and reports a balance of £42 on its accounts.

A CHEQUE for £300 has been handed over to the Peterborough Hospital, the result of the Hospital Saturday collection.

THE Royal South Hampshire Infirmary has just received a gift of £1,000 from Mr. A. Barlow, a resident in the county.

THE Countess of Zetland—so zealous in good works—has invited all the workhouse children in Dublin to a tea at the Viceregal Lodge.

THE new wing of the High Wycombe and Beaconsfield Memorial Cottage Hospital was formally opened by Mr. Coningsby Disraeli on the 19th ult.

A DOCTOR in Poland is reported to have been buried alive. Sounds were heard proceeding from the grave, but he perished before he could be disinterred.

THE EAST LONDON HOSPITAL FOR CHILDREN, SHADWELL, have received a present of cast linen from Her Majesty the Queen, and a present of toys from Her Royal Highness Princess Victoria of Teck.

VENTNOR has decided that it must have an infectious hospital. Previously it was hoped to combine with the hospital authorities of the whole island, but this scheme has fallen through, and Ventnor will stand by itself in the matter.

THE immediate vicinity of the Royal Military College, Sandhurst, seems to be especially attacked by influenza. In Windsor 1,500 cases are reported. At Dover appeals are made for assistance, so many are the sufferers amongst the poor.

THE LORD CARRINGTON has kindly consented to preside at the thirteenth festival dinner of the East London Hospital for Children, Shadwell, E., which will be held on 24th February (Wednesday), at the Whitehall Rooms, Hotel Métropole.

LADY FREDERICK CAVENDISH is about to inaugurate weekly entertainments for boys at the St. Martin's-in-the-Field Free Public Library. Her ladyship will lecture on travels in South Africa.

AS a result of a meeting of the medical men at Teignmouth, a resolution was read before the Local Board to the effect that an isolation hospital was absolutely necessary for the town. The Board have decided to refer the matter to the Finance Committee.

THE Lords Commissioners of the Admiralty have awarded the Greenwich Hospital Pension of £50 a-year, vacant by the death of Deputy Inspector-General of Hospitals and Fleets Andrew Murray, to Deputy Inspector-General of Hospitals and Fleets William H. Adam.

AN agricultural labourer named Woodford, and family, eight in all, ate tinned salmon, and the mother and six children became ill, but al recovered except one little girl, who died after a fortnight's illness. The deceased had been seen scraping the sides of the tin with a knife.

THE new buildings of the Wolverhampton Borough Hospital are now opened. The enlargement comprises two wards (male and female), each 50 ft. by 26 ft., contained in a pavilion building. Appertaining to each ward is a small single bed ward. The additions were designed by Mr. Berrington.

GEORGE BEALE, of Kilkenny, who was bitten by a mad dog two months ago, and who was afterwards treated at the Pasteur Institute, Paris, died from hydrophobia on Sunday night. Here is a statement for those who have been waiting and watching for a failure of the Pasteur cure.

A LOCAL hospital report supplies us with an amusing example of the popular idea of hospital treatment. A woman who applied for relief stated that she had been a patient at an infirmary, and there the diseases were cured by means of a piece of sticking plaster applied to the end of the nose.

THE Birmingham Oddfellows propose establishing a convalescent home, on the lines of the one possessed by the Ancient Order of Foresters, which has proved such a success. The Oddfellows number 50,000 in the Midland Counties, and these are to be asked to contribute 2d. per member a quarter.

A SORT of epidemic of hydrophobia is reported from Carcassonne, in the Commune of Pepieux. Near that town no fewer than forty dogs and cats, and even some sheep, have been killed as suspected of having hydrophobia. A man who was bitten by one of the supposed mad dogs has gone to Paris to be treated by M. Pasteur.

IT is reported from Moscow that the Governor, the Grand Duke Sergius, disguised himself as a peasant, and proceeded to several bakers' shops, to discover for himself the truth of the statements that the bakers were refusing to sell bread at the current prices. He found this was the fact, and summary punishment has been dealt.

THERE has been no individual more zealous this Christmas in adding brightness to the lot of the poor and sick than the Countess of Zetland. Amongst other kind deeds, she lent her presence at the fête at the Children's Hospital, Dublin, on the 28th ult. There were Christmas trees and other attractions, admired by the visitors and enjoyed by the little inmates.

THE new Cottage Hospital at Ledbury is of the Elizabethan style, and attached to it is a residence for a town nurse. Mr. Hadden, of Malvern, is the architect, and Mr. George Hill, of Ledbury, the builder. Although the building is not yet ready for the removal of the patients, the formal opening took place yesterday, when Lady Elizabeth Biddulph declared the building open.

MR. MICHAEL BIDDULPH, M.P., has given to the town of Ledbury, in commemoration of the coming of age of his eldest son, Mr. John Biddulph, a Cottage Hospital, at a total cost of about £3,000. The building occupies a commanding position in the town, and is immediately opposite a dwelling house in which the Ledbury Cottage Hospital was opened nearly twenty year ago.

THE principal distributions of the Birmingham Sunday Collection are as follows: General Dispensary, £668 16s. 10d.; Children's Hospital, £739 5s. 11d.; Eye Hospital, £676 12s. 5d.; Women's Hospital, £539 6s. 5d.; Deaf and Dumb Institution, £350 2s.; Blind Institution, £310 1s. 11d.; Homœopathic Hospital, £350 0s. 11d.; Orthopædic and Spinal Hospital, £297 15s.; Birmingham Sanatorium, £284 17s. 6d.; Lying-in Charity, £115 1s. 10d.

### THE STUDENT OF MEDICINE.

[All communications for this Supplement should be addressed to THE EDITOR of THE HOSPITAL, at 140, Strand, with the words, "Students' Column," written in the left hand top corner of the envelope.]

### APPOINTMENTS.

John A. Adams, B.A., M.B., R.U.I., appointed House Surgeon to the Halifax Infirmary and Dispensary, vice J. F. Woodyatt, L.R.C.P. Lond., M.R.C.S., resigned. T. H. Bishop, M.B., C.M., appointed House Surgeon Royal Portsmouth Hospital, Portsmouth. G. G. Clarke, M.R.C.S., L.R.C.P., appointed Assistant House Surgeon to the Halifax Infirmary and Dispensary, vice J. A. Adams, B.A., M.B. Arthur Templer Davies, B.A. Cantab., M.D., M.R.C.P. Lond., appointed Medical Officer to the Westminster and General Life Assurance Association. Stamford G. Felce, B.A., M.B., B.C.Cantab., appointed Assistant Medical Superintendent to the St. Saviour's Union Infirmary, East Dulwich, vice T. Fisher, M.D. Andrew Fullerton, M.B., B.Ch., B.A.O., reappointed Senior House Surgeon to the Miller Hospital and Royal Kent Dispensary, H. C. Garth, M.B., C.M., appointed Junior Resident Medical Officer to the Miller Hospital and Royal Kent Dispensary, vice C. Evans, resigned. H. Oswin Johnson, M.R.C.S., L.R.C.P., appointed House Surgeon to the General Infirmary at Gloucester and the Gloucester Eye Institution, vice J. L. Adam, M.B., C.M., resigned. D. O., Macgregor, M.D., appointed House Surgeon and Apothecary to the Northern Infirmary, Inverness. Francis George Penrose, M.D., M.R.C.P. Lond., M.R.C.S. Eng., appointed Medical Officer to the Westminster and General Life Assurance Association. Walter Ridley, M.B., M.S. Durh., F.R.C.S. Eng., appointed Honorary Surgeon to the Hospital for Sick Children, Newcastle-upon-Tyne. J. E. Thompson, M.B. Lond., C.M., M.R.C.S., appointed House Surgeon to the Wolverhampton Eye Infirmary. Walter Essex Wynter, M.D., B.S., M.R.C.P., F.R.C.S., appointed Assistant Physician to the Middlesex Hospital, vice J. K. Fowler, M.D.

### VACANCIES.

### Resident Medical Officers.

General Infirmary, Northampton.—House Surgeon—not under 23 years of age—Salary £125, with board, &c.

Holloway' Sanatorium Hospital for the Insane, Virginia Water.—Assistant Medical Officer—Salary £200, with board, &c.

### Non-Resident Medical Officer.

Dental Hospital of London.—Medical Tutor—Salary £40.

### NOTES FROM THE SCHOOLS.

### Guy's Hospital.

"THE GUY'S HOSPITAL GAZETTE."—The present number is enlarged to 24 pages, and contains several interesting articles.

### St. Thomas's Hospital.

"THE ST. THOMAS' HOSPITAL GAZETTE."—The present number contains an excellent portrait of Dr. Bristowe.

### PASS LISTS.

### University of London.

M.D. EXAMINATION:

MEDICINE.—J. O. W. Barratt, B.S., B.Sc., H. M. Bowman, W. H. Brazil, E. H. Brock, B.S., J. H. Bryant, B.S., H. E. Cuff, B.S., T. J. Dyall, B.S., P. C. Evans, J. Fawcett, B.S., W. H. R. Forsbrook, J. McD. Gill, R. F. Gill, T. A. Goodfellow, F. Grange, W. Griffiths, B.Sc., F. W. Hall, B.S., H. H. Horden, C. McG. Kitching, B.S., M. P. Ledward, R. Leigh, B.Sc., R. E. Lord, B.Sc., A. Lyndon, W. J. Maillard, R. D. Mothersole, M.S., E. S. Pasmore, E. P. Paton, T. L. Pennell, B.Sc. (gold medal), J. S. Richards, B.S., T. F. Ricketts, B.S., B.Sc., E. A. Roberts, J. Robertson, G. H. D. Robinson, B.S., A. G. Salmon, B.S., A. Scott, J. H. Sequeira, T. S. Short, E. H. Snell, B.Sc., S. H. Snell, B.S., W. T. Strugnell, S. A. Tidey, H. Tilley, B.S., H. W. Webber, B.S., L. Williams, B S., P. W. Williams, and E. Wills.

STATE MEDICINE.—D. S. Davies, G. H. O'Reilly, J. J. Ridge, M.D., B.S., B.A., B.Sc., and J. K. Warry.

### Naval Medical Service.

The following gentlemen who competed at the examination held on the 2nd inst. and following days at Examination Hall, Victoria Embankment, for appointment as Surgeons in the Royal Navy have been successful :—

| | Marks. | | Marks. |
|---|---|---|---|
| A. W. Sturdee ... ... | 2869 | C. S. Bennetts, B.A., M.B. ... ... ... | 2621 |
| E. M. Dubinson ... | 2699 | G. Ley ... ... ... | 2586 |
| A. G. W. Bowen, B.A., M.B., ... ... ... | 2684 | W. E. Marshall ... | 2514 |
| R. L. Price, M.B. ... | 2683 | M. L. B. Rodd... ... | 2500 |
| E. A. Shaw, B.A., M.B. | 2670 | H. L. Penny ... ... | 2478 |
| E. Cooper ... ... | 2669 | A. E. Kelsey, B.A., M.B. | 2473 |
| C. S. Facey, M.B. ... | 2633 | C. J. Fyfe, M.B. ... | 2463 |

